# “The effect of 48-weeks azithromycin therapy on levels of soluble biomarkers associated with HIV-associated chronic lung disease”

**DOI:** 10.1016/j.intimp.2023.109756

**Published:** 2023-03

**Authors:** Dan Hameiri-Bowen, Louis-Marie Yindom, Evgeniya Sovershaeva, Tsitsi Bandason, Justin Mayini, Andrea M Rehman, Victoria Simms, Lucky Gift Ngwira, Trond Flagestad, Tore Jarl Gutteberg, Grace McHugh, Rashida Abbas Ferrand, Sarah L. Rowland-Jones

**Affiliations:** a Nuffield Department of Medicine, University of Oxford, Oxford, United Kingdom; b UiT The Arctic University of Norway, University Hospital of North Norway, Tromsø, Norway; c Biomedical Research and Training Institute, Harare, Zimbabwe; d MRC International Statistics and Epidemiology Group, London School of Hygiene and Tropical Medicine, London, United Kingdom; e Liverpool School of Tropical Medicine, Liverpool, United Kingdom; f Malawi-Liverpool Wellcome Trust Clinical Research Program, Blantyre, Malawi; g Department of Clinical Research, London School of Hygiene and Tropical Medicine, London, United Kingdom

**Keywords:** Azithromycin, Immunomodulation, HIV, Lung Disease, Cytokines, CRP

## Abstract

**Objectives:**

HIV-associated immune activation contributes to chronic lung disease (CLD) in children and adolescents living with HIV. Azithromycin has immunomodulatory and anti-microbial properties that may be useful for treating HIV-associated CLD (HCLD). This study describes the effect of azithromycin on expression of plasma soluble biomarkers in children and adolescents with HCLD.

**Methods:**

This study was nested within a multi-site double-blind, placebo controlled, randomised controlled trial (RCT) of azithromycin in individuals aged 6–19 years with HCLD (defined as FEV1 z-score < -1) in Malawi and Zimbabwe (BREATHE (*NCT02426112****))***. Participants were randomized 1:1 to once-weekly oral azithromycin with weight-based dosing, for 48 weeks, or placebo. Twenty-six plasma soluble biomarkers were measured on a MagPix Luminex instrument at enrolment, after 48-weeks of treatment and 24-weeks after treatment cessation. Mixed effects models were constructed to compare biomarker expression across treatment and placebo groups.

**Results:**

Weekly azithromycin was associated with reduced levels of C-Reactive Protein (CRP), E-Selectin, Matrix metalloproteinase 10 (MMP-10). Treatment effects for all soluble biomarkers were not sustained 24-weeks after treatment cessation with biomarker expression returning to pre-treatment levels.

**Conclusions:**

We observed real-world effects of azithromycin on acute inflammation, neutrophil accumulation, and extracellular matrix degradation, that were not sustained after treatment cessation. These results are pertinent when using azithromycin for its immunomodulatory properties, or targeting pathways represented by the soluble biomarkers in this study.

## Highlights

• Azithromycin treatment reduced plasma levels of CRP, E-Selectin and MMP-10 in children with HIV-associated chronic lung disease.
• Limited immunomodulatory properties observed in large panel of soluble markers.
• No treatment effects were sustained after cessation of treatment.

## Graphical abstract

**Figure:**
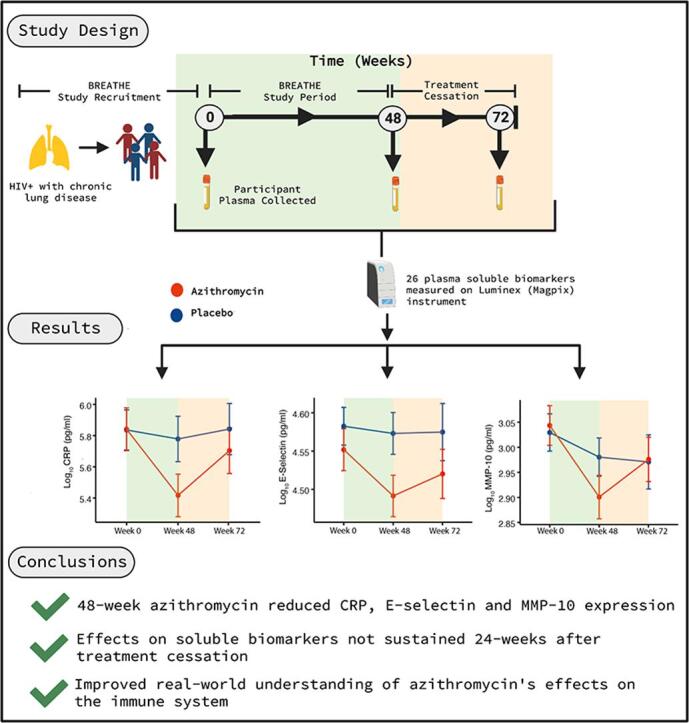

## 1 Introduction

Chronic lung disease in children with HIV is common despite antiretroviral therapy (ART) and its management is an important research priority [1], [2]. Studies have shown approximately 30 % of African children aged 10 years or more with perinatally-acquired HIV (PHIV) have HIV-associated chronic lung disease (HCLD). The clinical and radiological findings of HCLD are consistent with constrictive obliterative bronchiolitis (COB) as the predominant underlying cause of HCLD [3], [4]. The typical presentation is with chronic cough, hypoxia, breathlessness and reduced exercise tolerance with irreversible obstructive lung function defects, coupled with a picture of mosaic decreased attenuation on high resolution computed tomography [4], [5], [6], [7]. In the absence of effective treatment patients with HCLD are often erroneously treated for tuberculosis (TB) [8], [9].

Although the aetiology of OB remains poorly described, persistent immune activation, in part driven by HIV replication, alongside co-infections and microbial translocation, are thought to be important drivers of aberrant fibroproliferative remodelling and fibrosis of the small airways. Chronic inflammation is well described in the pathology of chronic respiratory diseases, and recent cross-sectional studies have reported associations between circulating markers of immune activation and HCLD in children with HIV [10], [11]. As such, treatments that reduce levels of systemic inflammation, or target plasma soluble markers associated with HCLD [10], [11], could be of benefit in individuals with HCLD.

Azithromycin is an oral macrolide antibiotic with a long half-life and high intracellular concentrations. Azithromycin is often used in the treatment of bacterial respiratory infection alongside chronic respiratory conditions such as bronchiectasis, interstitial lung disease and asthma [12]. Azithromycin is also reported to have potent immunomodulatory properties. Several studies have shown that azithromycin achieves therapeutic benefit in respiratory diseases, in part, owing to its immunomodulatory properties [13], [14], [15], [16]. *In vitro* studies have shown that azithromycin leads to proliferative inhibition, reduced Interferon gamma (IFN-γ) cytokine production and induction of apoptosis in bulk CD4 + T cells [17], [18], but the magnitude of this effect varies across T helper (Th) subsets [19]. Neutrophil accumulation and prolonged survival are characteristic of chronic airway diseases, where neutrophils drive cellular and tissue injury. Azithromycin likely leads to benefit in lung conditions by increasing the phagocytosis of apoptotic epithelial cells and neutrophils by macrophages [20], [21]. Azithromycin also exerts direct activity on airway epithelial cells, increasing epithelial barrier thickness *in vitro* [22], reducing mucus formation [23] and viral replication [24]. Azithromycin exerts immunomodulatory properties by inhibiting inflammatory cell signalling [25], reducing neutrophil accumulation, altering macrophage polarization [26], and supressing T cell activation by modifying the mTOR signalling pathway [18]. Owing to its strong association with persistent immune activation, these mechanisms suggest that the immunomodulatory effects of azithromycin would be of benefit to individuals with HCLD.

We recently completed a multi-site phase three randomised trial that assessed the effect of adjuvant treatment with 48-weeks weekly azithromycin on lung function in children and adolescents with HCLD – the BREATHE study (The Bronchopulmonary Function in Response to Azithromycin Treatment for Chronic Lung Disease in HIV-infected Children - ClinicalTrials.gov NCT02426112). Azithromycin was not associated with improved lung function (primary outcome) but individuals randomized to receive azithromycin had significantly lower hazards of respiratory exacerbations and hospitalisation (secondary outcomes) [27]. This group has previously described associations between plasma soluble biomarkers and the presence of HCLD, alongside the degree of airflow obstruction [11]. Consequently, prior to the trial, we hypothesised that improved lung function could be driven by azithromycin’s immunomodulatory effects. Although no effect on lung function was observed, the effect of azithromycin expression of plasma soluble biomarkers is important to describe, and could help us understand the epidemiological findings better.

In this paper we report the effect of 48-week treatment with azithromycin on the levels of 26 plasma soluble biomarkers in children and adolescents with HCLD. The biomarkers selected represent a range of immune system related and pathological pathways. The majority of biomarkers included were previously been associated with both HCLD and lung function in these groups [11]. Biomarkers represented pathways including systemic immune activation/inflammation (Beta-2-Microglobulin, Interferon induced protein-10 (IP-10), C-reactive Protein (CRP), Interferon Gamma (IFN-γ), sCD25, sCD27, sCD40-L, sCCL5), monocyte activation (sCD14, sCD163), Endothelial activation/cellular adhesion (E-Selectin, P-Selectin, Vascular cell adhesion molecule 1 (VCAM-1), Intracellular cell adhesion molecule 1 (ICAM-1), Vascular endothelial growth factor (VEGF)), coagulation (d-Dimer), apoptosis (Fas, Growth Differentiation Factor-15 (GDF-15), angiogenesis (Angiopoietin-1 (ANG-1) and extracellular matrix degradation (Matrix Metalloprotenaise-1,3,7,8,10,12) The levels of soluble biomarkers 24-weeks after cessation of treatment with azithromycin were further investigated.

## 2 Methods

### 2.1 Study design and participants

This study was nested within the BREATHE trial (*NCT02426112).* The trial protocol and findings are fully reported elsewhere [27], [28]. Briefly, BREATHE was a two-site, double blind randomised control trial which recruited children and adolescents aged 6 to 19 years with HCLD (defined as forced expiratory volume in 1 s (FEV_1_) *z* score < -1) who had been receiving ART for at least six months. Participants were recruited from outpatient HIV clinics in two public sector hospitals in Malawi and Zimbabwe. Participants were randomised 1:1 to intervention or placebo and followed at 24-week intervals. Weight-based oral azithromycin tablets (10-19·9 kg, 250 mg; 20-29·9kg, 500 mg; 30-39·9kg, 750 mg; > 40 kg, 1250 mg), or identical placebo tablets were provided. Plasma samples were collected at baseline and after 48-weeks. A subset of participants was followed up 24 weeks after the cessation of treatment (72-weeks).

### 2.2 Measurement of plasma soluble biomarkers

Soluble biomarkers were measured from cryopreserved plasma collected from heparinised blood samples at baseline, 48- and 72-week visits. Plasma aliquots were frozen and stored at −80 °C until use. The levels of all plasma soluble biomarkers were measured using Luminex multiplex bead arrays on a MagPix instrument according to the manufacturer’s protocol (Luminex technology, Hertogenbosch, Netherlands). Briefly, plasma samples were thawed on their first use, diluted appropriately and the levels of biomarkers were assessed immediately. Samples were run in duplicate, on a single MagPix instrument, with the mean of each technical replicate taken. Samples from individuals collected at all three timepoints were analysed together to minimise batch variation. Samples with measurement values falling outside the standard curve were repeated at appropriate dilutions. Those with levels consistently below limits of detection upon repeat were assigned half the minimum value measured for the specific biomarker under investigation. A full list of biomarkers and their abbreviations is provided in Supplementary Table 1.

**Table 1:** Demographic, anthropomorphic, and clinical characteristics of participants included in the analysis.

	Placebo (n = 170)	Azithromycin (n = 166)
Malawi Trial Site, n (%)	49 (28·8)	46 (27·7)
Zimbabwe Trial Site, n (%)	121 (71·2)	120 (72·3)
48 Week Data Available, n	154	157
72 Week Data Available, n	113	123
Age, Mean (SD)	15·3 (3·2)	14·6 (3·2)
Sex Male	82 (48·2)	88 (53·0)
Height for Age *z* score, Mean (SD)	−2·1 (1·2)	−2·2 (1·2)
Weight for age *z* score, Mean (SD)	−2·1 (1·5)	−2·3 (1·4)
Indicated previous treatment for Tuberculosis, n (%)	39 (22·9)	58 (35·2)
CD4 T Cell Count, Cells/mm^3^ (SE)	576·0 (354·9)	634·1 (386·8)
Supressed HIV-1 Viral load (<1000 copies/ml), n (%)	91 (53·5)	96 (57·8)
HIV-1 Viral load copies/ml, Median, IQR**	492·5 (13502·8)	331·0 (10961·0)
FEV_1_*z* score, Mean (SE)	−2·0 (0·7)	−2·1 (0·7)
FEV_1_:FEV Ratio, Mean (SE)	−0·8 (1·1)	−0·7 (1·1)
FEV Percent Predicted, Mean (SE)	73·1 (9·9)	72·5 (9·9)
Duration on ART, Mean Years (SE)	6·3 (3·2)	6·5 (3·3)
First Line ART Regimen – ATV/LPV/PI, n (%)	127 (74·7)	120 (72·3)
Second Line ART Regimen – EFV/NVP, n (%)	43 (25·3)	46 (27·7)

Table showing participant characteristics split by treatment arm. IQR = Interquartile Range, N = Number, ATV = Atazanavir, LPV = Lopinavir, PI = Protease inhibitor, EFV = Efavirenz, NVP = Nevirapine. FEV = Forced expiratory volume *One data point missing, ** Two data points missing, imputed with mean imputation.

### 2.3 Statistical analysis

Analyses were performed using R Studio (version 1.1.383). Principal component analysis (PCA) was applied to all available biomarker data points to understand changes in biomarker groupings over the trial. Principal components with eigenvalues > 1 were retained for analysis. Treatment effects on soluble biomarkers were assessed using the lme4 package in R [29]. Mixed effect models containing random intercept for site and slope per participant were constructed for each biomarker [30]. Two interaction terms of treatment group and dummy variables for 48-week and 72-week timepoints were included in the model. In the constructed models, the coefficient of treatment-time interaction term represents the intervention effect at that timepoint. Model covariates included age, sex, supressed HIV-1 viral load (<1000 HIV-1 copies/ml) and having previously been treated for tuberculosis. Results were adjusted for false discovery rate (FDR) using the Benjamini-Hochberg method [31].

### 2.4 Role of the funding source

The funders had no role in the study design, data collection, data analysis and interpretation or writing of the report.

## 3 Results

### 3.1 Participant characteristics

Full recruitment information is described elsewhere [27]. Three hundred and thirty-six of the 347 participants recruited into the trial were included in this analysis. Eleven participants were excluded from this analysis as they were incorrectly enrolled into the trial (FEV_1_ *z* score > -1). Characteristics of the study population by trial arm are presented in Table 1. Biomarker data were collected on all participants available (baseline (336), 48-weeks (311), 72-weeks (236). At baseline, there were no significant differences in biomarker titres between individuals in the azithromycin and placebo groups (Supplementary Table 1).

**Fig. 1:**
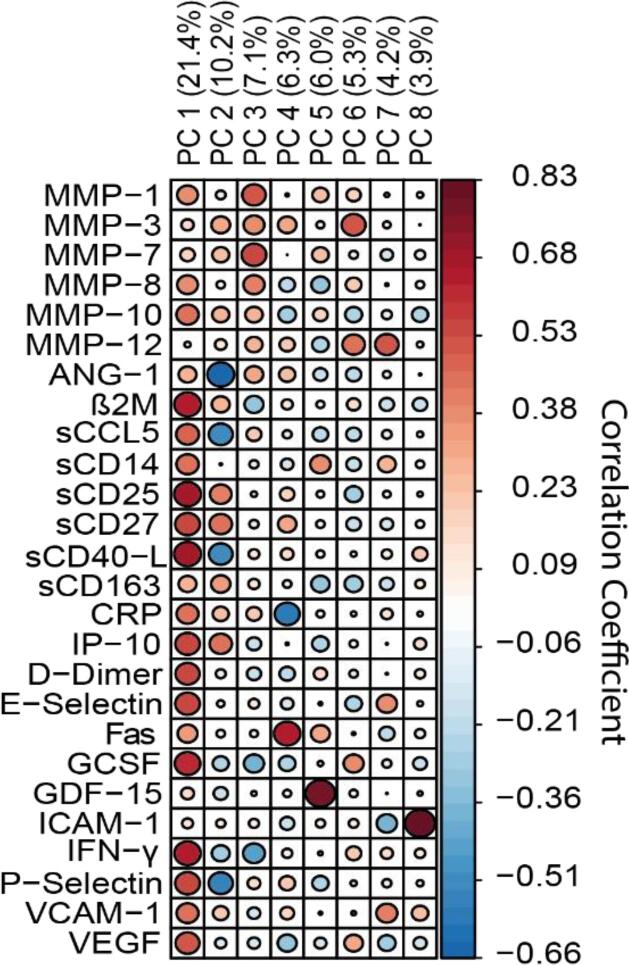
**Correlation between biomarkers and principal components derived from biomarker data.** Correlation is presented for each biomarker and principal components derived from PCA. Percentages represent proportion of variance explained by each component.

The correlations between plasma soluble biomarkers and principal components derived from biomarker data are shown in Fig. 1. Eight principal components had eigenvalues > 1 and were retained in downstream analyses. PC1 correlated with markers of immune activation (sCD25 sCD27, sCD40-L, IFN-γ, β2M). PC2 negatively correlated with ANG-1. PC3 consisted of several MMPs and PC4 positively associated with Fas and negatively correlated with CRP (Fig. 1).

### 3.2 Effect of azithromycin on levels of soluble biomarkers

**Fig. 2:**
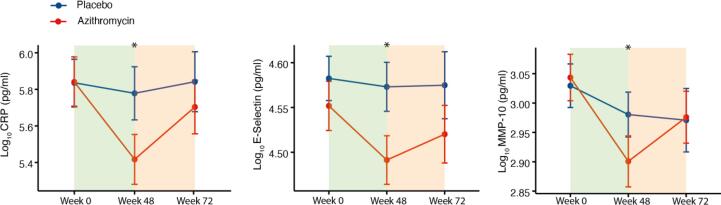
**Effect of 48-week azithromycin treatment on levels of soluble biomarkers.** Line plots showing baseline, 48-week, and 72-week biomarker levels split by trial arm. Only biomarkers with significant FDR adjusted p values in the regression model are shown. Asterisk signifies significant intervention effect as assessed in the regression model. Background colours represent the period receiving azithromycin (green) and period after cessation of azithromycin treatment (orange). Brackets around point represent 95% confidence interval of the mean. (For interpretation of the references to colour in this figure legend, the reader is referred to the web version of this article.)

At 48-weeks MMP-1, MMP-10, CRP, E-Selectin, GCSF and PC1, PC3 and PC7 all showed evidence of reduction in the azithromycin group (Table 2). Fas and PC4 were increased in the group receiving azithromycin. After FDR adjustment, CRP, E-Selectin and MMP-10 alongside PC3 and PC4 retained significance. Line plots of mean biomarker titre over time are presented in Fig. 2. 24-weeks after cessation of treatment, there was weak evidence for reductions in sCD14 and GCSF alongside increases in sCD163 in participants who had been randomized to azithromycin. All 72-week effects did not remain significant after FDR adjustment.

**Table 2:** Intervention effect of azithromycin treatment compared to placebo.

Biomarker (Log_10_)	48-week β ± SE	p Value	FDR Adjusted p Value	72-week β ± SE	p Value	FDR Adjusted p Value
MMP-1	−0·07 ± 0·03	0·016	–	−0·04 ± 0·031	–	–
MMP-10	**−0·09 ± 0·03**	**0·001**	**0·014**	−0·01 ± 0·031	–	–
MMP-12	−0·07 ± 0·03	0·023	–	−0·04 ± 0·04	–	–
sCD14	−0·01 ± -0·01	0.488	–	−0·03 ± 0·01	0·022	–
sCD163	0·02 ± 0·02	–	–	0·06 ± 0·02	0·008	–
CRP	**−0·37 ± 0·11**	**0·001**	**0·014**	−0·15 ± 0·12	–	–
E-Selectin	**−0·05 ± 0·02**	**0·002**	**0·02**	−0·02 ± 0·02	–	–
Fas	0·03 ± 0·01	0·016	–	−0·004 ± 0·01	–	–
GCSF	−0·04 ± 0·02	0·019	–	−0·04 ± 0·02	0·03	–
PC 1	−0·56 ± 0·23	0·014	–	−0·42 ± 0·25	–	–
PC 3	**−0·32 ± 0·11**	**0·003**	**0·022**	−0·12 ± 0·12	–	–
PC 4	**0·37 ± 0·11**	**0·001**	**0·014**	0·02 ± 0·13	–	–
PC 7	−0·18 ± 0·08	0·022	–	−0·13 ± 0·09	–	–

Intervention effect on biomarkers (log_10_) and principal components (PC). Coefficients (β) represent interaction between trial arm and time at 48 weeks and 24 weeks post treatment cessation (72 weeks). HIV-1 suppression, age, sex, and indication of previous treatment for TB are included as covariates. P values represent significance of biomarker and dummy-time value interaction term in mixed effect model. FDR adjusted p-value calculated using Benjamini-Hochberg method. Only biomarkers with p < 0·05 at any timepoint shown. Blank cells are non-significant (p > 0.05). SE = Standard Error.

## 4 Discussion

The immunomodulatory effect of azithromycin has been of particular interest and importance in treatment of chronic inflammatory viral diseases [32]. Our study adds evidence about the real-world properties of azithromycin, enriching the clinical understanding and potentially develop our understanding of epidemiological findings from the trial. Previous reports have shown that azithromycin reduces the recruitment of neutrophils, macrophages and other phagocytes to the site of inflammation [12]. Azithromycin also supresses nuclear factor-kappa B [32] and mTOR activity, leading to the inhibition of cytokine secretion by bulk CD4 + T cells [18], but more recent reports have described different magnitudes of effect in different T helper cell subsets [19]. In the context of HCLD, the immunomodulatory effect of azithromycin has the potential to reduce HIV-associated pathogenesis driven by persistent immune activation.

We report that 48-week azithromycin treatment was associated with reduced circulating levels of CRP, E-Selectin and MMP-10. A principal component characterised by markers of extracellular matrix degradation was decreased, and a component strongly correlated with soluble markers of apoptosis was increased. The most striking effect was on circulating levels of CRP. CRP is an IL-6 dependent protein with a short half-life produced in the acute phase of bacterial, fungal, and viral infection. Local and systemic CRP are involved in the development and maintenance of a variety of lung pathophysiological conditions [33], and increased levels have been associated with higher rates of FEV_1_ decline [34]. Reductions in CRP have previously been observed following three months of azithromycin treatment in adults with cystic fibrosis [35]. CRP is associated with increased mortality during treated HIV-1 infection, independent of CD4 + T cell count [36], and is elevated in individuals initiating ART 2–3 weeks after HIV-1 infection [37]. Previous studies have shown that high or increasing levels of CRP indicate antibiotic treatment failure or the development of infective complications [38]. In our study, reduced CRP could reflect reduced acute infections in participants randomised to azithromycin, which itself is a driver of inflammation. Recent reports from Abotsi *et al.* have described reduced carriage of common respiratory bacteria following azithromycin treatment in this cohort [39]. We hypothesise that these findings could be linked, with CRP mirroring reduced in bacterial infections in this cohort.

Reduced levels of neutrophil adhesion molecules like E-selectin - required for the extravasation of neutrophils from blood vessels into the interstitial space [32] – could represent attenuated neutrophil infiltration and accumulation in participants receiving azithromycin. Azithromycin increases apoptosis of neutrophils, preventing the damaging consequence of neutrophil accumulation by reducing the migration of neutrophils to sites of inflammation. Attenuation of damaging neutrophil driven responses could also be achieved by azithromycin’s effects on the coordination of inflammation and repair by macrophages (reviewed in Venditto et al., [40]).

Evidence that azithromycin is exerting an effect through inhibiting neutrophils is supported by lower expression of GCSF in participants receiving azithromycin (prior to FDR adjustment). GCSF can delay apoptosis and promote longer life spans of neutrophils at sites of inflammation [41]. Delayed apoptosis and prolonged neutrophil survival slows resolution of acute inflammation and increases leakage of damaging content from dying cells [42]. This mechanism is consistent with the increase in PC4, which is strongly correlated with expression of the apoptosis marker Fas. In a previous study, Lei *et al.* reported that azithromycin-associated downregulation of serum GCSF was able to achieve therapeutic effect in children with reactive airway disease [43]. Condliffe *et al*. further showed that neutrophil elastase, produced in the context of neutrophil accumulation, could drive tissue destruction in chronic obstructive pulmonary disease [44]. These findings suggest that reduced neutrophil accumulation could be beneficial in this cohort.

We reported a reduction in MMP-10 in the group receiving azithromycin. PC3, composed of a combination of MMP-1, MMP-3, and MMP-7, was also reduced. Azithromycin increases the structural integrity of bronchial epithelial cells *in vitro* through improved barrier formation during differentiation, improved maintenance of an established barrier and improved response to damage [22]. Increased cell integrity is accompanied by attenuated expression of inflammatory markers including MMPs [12]. MMPs have varied roles, including both degradation and remodelling of the extracellular matrix (ECM) during tissue repair and regulation of cell proliferation, adhesion, migration, and apoptosis. Reduced breakdown of the pulmonary ECM is thought to give less opportunity for repair with consequent fibrosis and abnormal remodelling of the small airways. In keeping with this, elevated levels of MMPs have been associated with multiple lung pathologies including OB [45]. The exact significance of elevated MMP-10 levels is poorly described. Ostridge *et al.* report an association between MMP-10 with markers of small airway disease assessed by computerised tomography, and suggest that MMP-10 may play a significant role in the development of small airway remodelling and associated airflow obstruction [46]. We have previously reported an association between MMP-10 and both the presence of HCLD and the extent of airflow obstruction in a cross-sectional analysis of this cohort [11]. Modest reductions in several MMPs following azithromycin treatment *in vitro* and *in vivo* have previously been described [47], [48]. The findings presented are consistent with the reduction in PC3 as a general marker of several MMPs. This finding suggests that azithromycin may have broad effects on MMP production, targeting a common pathway in the expression of all these proteins. As MMPs are regulated by NF-kB and AP-1, azithromycin’s modulation of promoter binding is a probable mechanism of effect [49].

We report no evidence of sustained effects on plasma soluble biomarkers 24-weeks after the cessation of treatment. Prior to FDR adjustment, the inverse effects of treatment on levels of sCD14 and sCD163, both markers of monocyte activation are of particular interest. Azithromycin inhibits the transcription factor AP1 and reduces lipopolysaccharide-induced cytokines such as sCD14, a marker of microbial translocation. Microbial translocation drives systemic immune activation in people living with HIV [50]. Increased circulating sCD163 is suggestive of M2 polarization of macrophages, which azithromycin has been shown to promote [51]. A shift towards M2 macrophages in azithromycin-treated individuals would reduce systemic inflammation and promote wound healing and repair. As the only signal persisting after treatment cessation, further work aimed at understanding the long-term effects of azithromycin on macrophages would be highly informative. Moreover, as the half-life of azithromycin is around 6 days, future studies involving more regular sampling after cessation of treatment could help us understand how long the immunomodulatory effects of azithromycin on plasma soluble biomarkers lasts. Developing this understanding is key when weighting the potentially beneficial immunomodulatory properties of azithromycin use against the development of antimicrobial resistance.

Despite the effects on MMP-10, CRP and E-Selectin, the BREATHE trial reported no significant effect of azithromycin on lung function. This result is particularly surprising considering CRP, E-selectin and MMP-10 were associated with increased odds of HCLD and lower FEV_1_ *z* score in participants with HCLD [11]. There could be several reasons for these findings. Firstly, improvements in FEV_1_ are hard to achieve. The immunomodulatory properties described may indicate that azithromycin could be acting to reduce the rate of decline of FEV_1_ *z* score, but a longer trial period may be required for adequate power to detect a difference between the two groups. Secondly, although associated with FEV_1_ *z* score, the biomarkers altered by azithromycin treatment may not be of central importance for HCLD progression. Attia *et al.* previously reported strong associations between markers of monocyte activation and HCLD, and our group previously identified the central association of T cell activation markers such as sCD25 and sCD40-L with the HCLD described in this cohort. Treatment options targeting these pathways may be more effective at reducing lung pathology driven by immune activation. In this study, attenuation of the damaging consequences of neutrophil accumulation, bacterial co-infection and aberrant extracellular matrix remodelling may be too little too late to affect an outcome like FEV_1._

As it is increasingly realised that comprehensive HIV care should include diagnosis and management of comorbidities. As a safe and well tolerated antibiotic that has previously been reported to have immunomodulatory properties, azithromycin is an attractive treatment option. These findings confirm effects of azithromycin on markers associated with inflammation, neutrophil accumulation, and extracellular matrix degradation in real world settings. Our results, indicating that the immunomodulatory effect of azithromycin was not sustained 24 weeks after treatment is important for understanding the longer-term properties of azithromycin treatment. These findings raise important considerations for azithromycin use in the treatment of HIV-associated comorbidities, alongside other settings. Moreover, the soluble biomarkers affected by azithromycin were not key drivers of HCLD in this population. Consequently, use of azithromycin for treating immunopathogenic HIV-associated comorbidities, and specifically HCLD, should be carefully reviewed based on mechanisms thought to be driving pathologies.

The strengths of this study include the high rates of follow up and completeness of the biomarker data. BREATHE participants were randomised to either azithromycin or placebo, thereby controlling for unmeasured differences between participants. Biomarker measurements were all performed in duplicate, on the same instrument, and all participant time points were run in parallel to minimise batch effects. The study has several limitations, primarily the fact that sustained adherence to azithromycin over a year is challenging for children and adolescents, however, this represents real world effects and contributes to generalizability of these findings. The generalizability of these findings are also reduced by the specific population (older children and adolescents with a potentially unique aetiology of HCLD). Consequently, these results should not be extrapolated to other age groups where the drivers of HCLD may differ. Although CRP was measured, markers such as IL-6 and immune-activation markers such as TNF would have been informative for interpretation of the results presented. Pilot studies excluded these markers based on their difficulty of detection in this cohort. Although loss-to-follow-up was low between baseline and 48-weeks, a substantial number of individuals were not included in the 72-week timepoint due to withdrawal, death, and loss to follow up. Although the characteristics of the two arms were no different at 72-weeks, there is a potential for unmeasured non-random loss to follow up in this study. Future studies, and studies employing similar methods to this study should include examine cell populations in the peripheral blood. Without this data it remains hard for us to ascribe the findings presented to the direct actions of azithromycin.

In conclusion, we describe several effects of azithromycin on levels of plasma soluble biomarkers that are consistent with its known mechanisms of action. Plasma soluble biomarkers involved in neutrophil accumulation, response to bacterial infection, and extracellular matrix degradation were reduced in participants receiving azithromycin. The signals reported were reversible following cessation of treatment, increasing confidence that these effects are owing to the action of azithromycin. The weak signal of long-term immunomodulation of macrophages fits previously described effects of azithromycin on M2 polarisation and should be further explored. Interestingly broad immunomodulatory effects on pro-inflammatory biomarkers, previously strongly associated with lung function in this cohort, were not observed. As with all anti-microbial agents, their benefits should be weighed against the potential to drive antimicrobial resistance. Advancing our real-world understanding of the immunomodulatory impact of azithromycin allows us to make better informed decisions on when azithromycin treatment may be best used.

## Funding

This work was supported by Research Council of Norway, through their GLOBVAC programme, with additional support from Helse Nord. RAF is funded by the Wellcome Trust through a Senior Fellowship in Clinical Science. DHB is funded by the Wellcome Trust PhD Programme in Genomic Medicine and Statistics (Wellcome award number 108861/B/15/Z). AMR & VS are additionally funded by the UK Medical Research Council (MRC) and the UK Department for International Development (DFID) under the MRC/DFID Concordant agreement, also part of the EDCTP2 programme supported by the European Union, Grant Ref: MR/R010161/1. The graphical abstract was created using BioRender.

This research was funded in whole, or in part, by the Wellcome Trust 108861/B/15/Z. For the purpose of Open Access, the author has applied a CC BY public copyright licence to any Author Accepted Manuscript version arising from this submission**.**

## Declaration of Competing Interest

The authors declare that they have no known competing financial interests or personal relationships that could have appeared to influence the work reported in this paper.

## Data Availability

Data will be made available on request.
